# Exploring the diffusion, application, and scalability of entrustable professional activities beyond medical education: A scoping review protocol

**DOI:** 10.1371/journal.pone.0330549

**Published:** 2026-10-22

**Authors:** Anjali Jagannathan, Julia Micallef, Otto Sanchez, Adam Dubrowski

**Affiliations:** Faculty of Health Science, Ontario Tech University, Oshawa, Ontario, Canada; PLOS: Public Library of Science, UNITED KINGDOM OF GREAT BRITAIN AND NORTHERN IRELAND

## Abstract

Competency-based education (CBE) is gaining prominence in post-secondary education due to its effectiveness in optimizing workforce readiness. However, assessing competence and workforce readiness presents a significant challenge. Entrustable professional activities (EPAs) offer a promising solution by translating competencies into observable, entrustable tasks. While EPAs have been widely adopted in clinical training, their application in non-clinical educational contexts remains minimal. Guided by Rogers’ Diffusion of Innovation theory, this scoping review aims to map the diffusion of EPAs beyond medical education and examine their scalability to non-clinical educational contexts, where real-world practice errors may undermine system integrity, educational outcomes, or public trust. This review will progress through five stages following Arksey and O’Malley’s framework. Searches will be conducted across Ovid MEDLINE, CINAHL, Scopus, ERIC, and Google Scholar. The research question is: “How have EPAs been adapted, developed and implemented outside of medical education, and what factors influence their adoption in non-clinical educational contexts?” The search will include studies that describe, evaluate, or reflect on the adaptation, development, or implementation of EPAs outside medical education, and exclude studies that only propose, define, or discuss EPAs, focus on medical education, or are not published in English. Titles and abstracts will be reviewed first, followed by the full-text examination of relevant studies. Data will be extracted and summarized to highlight key findings. A two-reviewer protocol will ensure quality during screening and data extraction. The findings from this review may provide practical guidance for faculty, curriculum developers, institutional administration, industry partners, and accrediting or policy bodies involved in curriculum and assessment framework design. Further, the findings may inform the rationale for scaling EPA-based assessment frameworks to non-clinical educational contexts and offer recommendations to guide their adaptation, development, and implementation, addressing CBE assessment challenges and creating a replicable model for future non-clinical program development.

## Introduction

Competency-based education (CBE) is gaining prominence in higher education due to its emphasis on demonstrated competence, with increasing empirical support for its effectiveness in optimizing workforce readiness [1]. However, while competency frameworks outline the knowledge, skills, and attitudes (KSAs) essential for professional practice, KSAs remain intrinsic attributes that cannot be directly observed in real-world performance [2]. Hence, conducting reliable, defensible assessments of competence and workforce readiness remains a persistent challenge undermining CBE [2]. In medical education, Ten Cate [3] addressed this challenge by introducing Entrustable Professional Activities (EPAs), which operationalize competencies as observable, measurable, and entrustable tasks. The strength of EPAs lies in their ability to shift the focus of assessment from isolated KSAs to the real-world execution of professional activities, bridging the gap between competency frameworks and professional practice [2]. In medical education, EPA proficiency is developed longitudinally across curricula aligned with Miller’s competency assessment model [4], where learners progress from classroom-based education to simulation training, to real-world performance [2], with assessments embedded at each stage. This structure can support the development of both canonical competence (the knowledge and skills required to perform EPAs) and contextual competence (the ability to integrate and apply KSAs to dynamic real-world practice situations) [5]. Within this structure, EPAs enable entrustment-driven assessment, in which trainees are progressively granted autonomy as they develop proficiency [2], and workforce readiness upon graduation.

Despite their demonstrated efficacy and widespread implementation in medical education [6], EPAs remain largely confined to clinical training programs that lead to licensure in medicine, nursing, or allied health professions (e.g., dietetics, physiotherapy, pharmacy etc.). These programs are typically accredited and include formal clinical placements as part of their curriculum. While there have been initial efforts to define EPAs for non-clinical roles (e.g., translational scientists, health professions educators, simulation leaders, residency program directors, graduate students in the health professions) [7–13], the scaling and application of EPAs in these domains has been minimal. Real-world practice errors in these domains may not directly harm patients, as is the case in clinical professions, but can undermine system integrity, educational outcomes, or public trust. As such, EPA-based assessment frameworks present a compelling opportunity for adaptation in non-clinical educational contexts. However, successful implementation will require careful contextual adaptation, accounting for institutional policies, supervision and autonomy structures, and the perspectives of key involved parties, including faculty, curriculum developers, administration, industry partners, and accrediting or policy bodies. This underscores the importance of examining the barriers and facilitators that influence the adoption of EPAs in non-clinical educational contexts.

To contextualize the diffusion of EPAs in non-clinical educational contexts, it is important to distinguish the structural and cultural features of clinical versus non-educational contexts. Rogers’ Diffusion of Innovations (DoI) theory [14] provides a conceptual framework for understanding how innovations spread through systems over time, identifying core factors that enable or constrain their successful adoption: (1) relative advantage, the perceived benefits over existing approaches; (2) compatibility, the alignment with needs and values; (3) complexity, the perceived ease of understanding, utilization, and implementation; (4) trialability, the degree to which the innovation can be tested and piloted; and (5) observability, the visibility of the benefits and results [14]. Table 1 outlines key dimensions differentiating clinical and non-clinical educational contexts in relation to these core factors. This comparison illustrates how EPAs, originally designed for medical education [3], can be adapted to non-clinical educational contexts.

**Table 1:** Comparison of clinical and non-clinical educational contexts for entrustable professional activities.

Dimension	Clinical Education Environments	Non-Clinical Education Environments	Implications for Adapting EPAs to Non-Clinical Educational Contexts
Primary Purpose of Training	Preparation for licensure and safe patient care through direct clinical practice.	Preparation for technical, educational, operational, or coordination roles supporting education, research, or operations.	Assessment should emphasize workforce readiness to safeguard system integrity and public trust.
Nature of Risk and Responsibility	High stakes: Errors directly impact patient safety and care quality.	Indirect or systemic stakes: Errors may affect system integrity, public trust, educational quality, or institutional outcomes.	Entrustment thresholds should be adapted to reflect performance abilities rather than patient safety.
Supervision and Autonomy	Gradual reduction of supervision as learners demonstrate clinical proficiency, ultimately leading to summative EPA-entrustment decisions.	Varied supervision models depending on task complexity and institutional policies.	EPA-based assessment frameworks should define levels of autonomy relevant to the role (e.g., equipment operations, data management).
Training Environment	Primarily in clinical placements or simulated patient encounters.	Academic, laboratory, or workplace-based simulation and applied project settings.	EPAs should be assessed in simulation and work-integrated learning curricular components aligned with professional workforce demands.
Regulatory and Accreditation Frameworks	Guided by professional colleges, accreditation bodies, and regulatory standards.	Often lacking clear regulatory oversight; institutional or program-level governance.	EPAs can help establish standardization and accountability where none exists.
Professional Identity Formation	Anchored in clinical expertise, patient advocacy, and ethical practice.	Emerging identities centered on technical, educational, and operational expertise.	EPA-based assessment frameworks should support progressive autonomy and integration into the professional community in non-clinical educational contexts.
Diffusion Challenges	High compatibility and observability; entrenched in medical culture.	Lower compatibility and observability; requires contextual adaptation and buy-in from involved parties.	EPA scaling and adoption strategies should emphasize relative advantage, visibility of outcomes, and pilot testing.

As shown in Table 1, while clinical and non-clinical education differ in stakes, supervision structures, and regulatory environments, EPAs offer a promising solution to CBE assessment challenges in both domains, as the underlying logic of entrustment is broadly applicable to any field where real-world performance carries risk. Non-clinical educational contexts can enhance compatibility and observability by adapting EPAs to reflect their own measures of professional trust and accountability, such as technical or operational accuracy, educational quality, or procedural safety. Framing EPAs in this way not only reduces perceived complexity, but also increases the relative advantage of adoption in new contexts. Consequently, adapting EPA frameworks in non-clinical educational contexts may support scalable, CBE assessment models that bridge the gap between theory and practice, fostering workforce-ready graduates.

Given the limited literature on EPAs outside medical education, a scoping review is the optimal methodology to explore their scalability to and application in non-clinical educational contexts [15]. While previous scoping reviews have explored the use of EPAs across clinical disciplines [16,17], to our knowledge, no review has mapped their diffusion or assessed their scalability to non-clinical educational contexts. Thus, informed by Rogers’ DoI theory [14], this review will not only map the diffusion of EPAs beyond medical education, but also assess the contextual factors that affect their scaling and application in non-clinical educational contexts, highlighting lessons learned from early adopters, uncovering strategies for successful adaptation, development, and implementation, and determining where EPAs may most appropriately diffuse in the future. If the review confirms the scarcity of existing literature on EPAs in non-clinical educational contexts and evidence of their transferability, it will inform the rationale for adapting, developing and implementing EPA-based assessment frameworks within these contexts. The research question explored through this scoping review will be: “*How have EPAs been adapted, developed, and implemented outside of medical education, and what contextual factors influence their adoption in non-clinical educational contexts?”*

## Methods

### Ethics statement

Given that the review will exclusively rely on analysis of publicly available materials with no human participants or data collection being conducted, no ethics approval is required.

### Protocol design

This scoping review will be guided by the methodological framework proposed by Arksey and O’Malley [18], which outlines five-stages: (1) identifying the research question; (2) identifying relevant studies; (3) selecting studies for inclusion; (4) charting the data; and (5) collating, summarizing, and reporting the results. To ensure methodological quality, the review will also draw upon the Joanna Briggs Manual for Evidence Synthesis [19] and the results will be reported in accordance with the Preferred Reporting Items for Scoping Reviews and Meta-Analysis Protocols (PRISMA-ScR) Checklist [20] presented in the S1 Checklist. Additionally, a health science librarian will be consulted to support database selection and search strategy development. As of April 2026, stage 1 and 2 have been completed, having identified the research question, developed a search strategy, selected databases, and identified relevant studies in consultation with a health sciences librarian. The following subsections describe each stage in detail, outlining the rationale, scope, and intended outputs.

### Stage 1: Identifying the research question

Guided by Rogers’ DoI theory [14], the primary objective of this scoping review is to explore the existing literature to determine where and how EPAs are used beyond medical education and examine contextual factors that influence adaptation, development, and implementation in non-clinical educational contexts. In consultation with the research team, the following research question has been developed: “*How have EPAs been adapted, developed and* *implemented beyond medical education, and what contextual factors influence their adoption in non-clinical educational contexts*?”. To address this question, the specific aims of the proposed scoping review are (1) to map the distribution and contexts in which EPAs have been adopted beyond medical education (diffusion); (2) to characterize the adaptation, development, and implementation approaches used to operationalize EPAs across these contexts (application); (3) to examine the reported conditions and indicators of transferability (scalability) of EPAs to non-clinical educational contexts. The findings of this review will be interpreted through the lens of the five core attributes identified in Roger’s DoI theory [14], aiming to identify the contextual factors that enable or constrain the adoption of EPAs in non-clinical educational contexts. If the review confirms the scarcity of EPAs in non-clinical educational contexts and evidence of scalability aligned with DoI factors, it will inform the rationale and provide insights to guide their adaptation, development, and implementation in non-clinical educational contexts, while also determining where EPAs may most appropriately diffuse in the future.

### Stage 2: Identifying relevant studies

Four literature databases, covering the medical, nursing, science and technology, and education fields, will be searched: Ovid MEDLINE, CINAHL, Scopus, and ERIC. Key concepts derived from the research question will be used to create free-text codes for the search strategy on Ovid MEDLINE, which will then be translated for the remaining three databases. The search strategy will include keywords such as “entrustable professional activities”, “entrustment”, “health professions education,” “health sciences education”, “non-clinical program”, “educator”, “facilitator” and their synonyms, and a search filter developed at the University of Alberta will be modified and used to derive studies related to clinical trainees and roles outside medical education, using terms such as “nursing,” “pharmacy”, “audiology”, “public health”, “residency”, “internship”, “trainee”, and their synonyms [21]. These free-text codes will be combined with controlled subject headings specific to each of the four literature databases to formulate the search strategy on each. The detailed search strategy for Ovid MEDLINE is presented in the S2 Table. To explore innovative, emerging results beyond published literature, Google Scholar will also be searched using the following search string: (“entrustable professional activities” OR entrustment) (“non-clinical program” OR “health professions education” OR healthcare OR dental OR nursing OR pharmacy OR premedical OR public health OR healthcare OR audiology OR chiropractic OR dietetics OR midwifery OR optometry OR paramedic OR psychology OR technology OR therapy OR veterinary OR facilitator OR director OR educator OR teacher). The first 500 results will be screened. This search strategy was developed in consultation with a health science librarian in accordance with the Peer Review of Electronic Search Strategies (PRESS) guidelines [22].

No time limits will be applied to the search strategy. While EPAs were formally introduced by Ten Cate in 2005, this review seeks to identify not only how and where EPAs have been developed and implemented beyond medicine, but also the contextual factors that enable or constrain their adoption to non-clinical educational contexts. Removing temporal restrictions allows for the identification of conceptual antecedents that may align with the principles of EPAs and highlight lessons learned from early efforts to develop and implement similar entrustment-based assessment frameworks, thereby providing a more comprehensive understanding of the barriers and facilitators influencing EPA adoption across non-clinical educational contexts. Furthermore, the reference lists of relevant studies will be manually searched to identify any new studies that address the research question.

### Stage 3: Study selection

Initially, EndNote 21 [23] will be used to screen the selected studies and eliminate duplicates. The results will then be imported into EPPI-Reviewer software [24] for further screening and selection. The studies will be selected based on a specific inclusion and exclusion criteria to ensure they address the research question. Screening will follow a two-step process. In step 1, two reviewers will independently screen a subset of titles and abstracts (10%) to calibrate application of the eligibility criteria. A minimum threshold of ≥ 90% will be established a priori [25]. If ≥ 90% agreement is achieved, the primary reviewer will screen the remaining studies, with any uncertain cases referred to the second reviewer for discussion and consensus. If ≥ 90% agreement is not achieved, disagreements will be discussed and the eligibility criteria will be clarified or refined as needed, after which both reviewers will independently screen an additional subset of titles and abstracts (10%) and repeat the calibration process until ≥ 90% agreement is reached [25]. Any unresolved disagreements will be resolved by consulting a third reviewer. In step 2, both reviewers will independently screen 100% of the remaining studies in full text. Any disagreements will be resolved through constructive discussion until consensus is reached. If consensus cannot be reached, a third reviewer will be consulted. Eligibility will be determined based on the extent to which sources provide extractable information on EPA adaptation, development, or implementation, rather than solely on publication type.

The inclusion criteria will include a need for the study to: (1) describe, evaluate, or reflect on the adaptation, development, or implementation of EPAs in educational contexts outside medical education; and/or (2) report empirical findings or practice-based insights, including barriers, facilitators, outcomes, or lessons learned. Study designs may include empirical studies (qualitative, quantitative, mixed-methods, program evaluations, and practice-based commentaries), provided they include substantive description or analysis of implementation. The review will exclude criteria studies that: (1) only propose, define, or conceptually discuss EPAs without describing their adaptation, development, or implementation; (2) focus exclusively on EPAs in medical education; or (3) are not published in English. Conference abstracts and posters will also be excluded due to their limited methodological and contextual detail, which are required for data extraction and analysis. The study selection process will be reported using a PRISMA flow diagram.

### Stage 4: Charting the data

EPPI-Reviewer [24] will be used to extract and chart the data, and the study title, author details, country of origin, study design, educational context in which EPAs are being used, learner characteristic, study aims, strategies employed for their adaptation, development and implementation, study outcomes/conclusions, contextual factors influencing scaling and adoption in non-clinical educational contexts aligned with DoI factors [14] and any other key findings related to the research question (i.e., outcomes, recommendations for adaptation, development, or implementation, and implementation barriers and facilitators) will be extracted.

### Stage 5: Collating, summarizing, and reporting the results

The context in which EPAs were adopted, the strategies used for adaptation, development or implementation, and reported conditions and indicators of transferability (scalability) of EPAs to non-clinical educational settings aligned with Rogers’ DoI factors [14], will be reported. Both qualitative and quantitative data will be descriptively summarized in-text and presented in graphs and tables where appropriate, adhering to the Joanna Briggs Institute Manual for Evidence Synthesis and the PRISMA-ScR Checklist [20].

## Quality assurance

Several measures will be taken to ensure quality. First, EndNote 21 [23] will be used to organize the studies identified in stage 2 and create references for those that meet the inclusion criteria. Then, EPPI-Reviewer [24], will be used in stage 3 to screen and select studies. In stage 3, two reviewers will independently screen a subset of titles and abstracts (10%) to assess eligibility and ensure calibration of the application of inclusion and exclusion criteria. If ≥ 90% agreement is not achieved, the eligibility criteria will be clarified or refined as needed and the process will be repeated as needed until ≥90% agreement is reached. Then, the primary reviewer will screen the remaining studies, referring any uncertain cases to the second reviewer, and consulting a third reviewer if consensus cannot be reached [25]. During the full-text screening stage, both reviewers will screen 100% of the studies to assess eligibility, resolve disagreements through constructive discussion, and consult a third reviewer if consensus cannot be reached.

Additionally, during the data extraction and charting process in stage 4, one reviewer will extract the data from 100% of the studies and a second reviewer will independently extract data from two random samples (5% each) of the remaining studies to maintain consistency and methodological rigour. Disagreements will be resolved through discussion between the two reviewers, and a third reviewer will be consulted if necessary. Any such disagreements and their resolutions will be reported to ensure transparency. In cases of missing data, attempts will be made to contact the study authors for clarification. If the missing information cannot be obtained, this will be documented and the implications for data synthesis will be noted.

## Dissemination

The findings of this scoping review hold the potential to inform future research and practice in expanding EPAs further beyond their medical education origins and offer insights to guide the development and implementation of EPA-based assessment frameworks within non-clinical educational contexts. Aligning framework design with the contextual realities of non-clinical educational contexts can guide institutions in developing scalable, competency-based assessment models that balance academic rigor with workforce readiness. The estimated date of the review’s completion is in September 2026. The results will be submitted for publication in a peer-reviewed journal, contributing to and guiding future advancements in the broader field of EPA-based assessment and CBE. The findings will be available only after this scoping review is conducted and completed.

## Strengths and limitations

This scoping review protocol possesses several notable strengths. It represents the first effort to explore how EPAs have been developed and implemented beyond their origins in medical education, inclusive of non-clinical educational contexts. The review employs a comprehensive search strategy across four major literature databases (Ovid MEDLINE, CINAHL, Scopus, and ERIC) supplemented with grey literature (Google Scholar) to maximize comprehensiveness. The consultation with a health sciences librarian and application of PRESS guidelines [22] ensure methodological rigor and transparency. The study design adheres to the methodological framework proposed by Arksey and O’Malley [18], the Joanna Briggs Institute Manual for Evidence Synthesis [19], and the PRISMA-ScR checklist [20], enhancing reproducibility and credibility. Quality assurance measures, including a screening protocol involving two independent reviewers, establishing a minimum threshold of ≥ 90% a priori, and the use of both the EndNote 21 [23] and EPPI-Reviewer software [24], further strengthen methodological rigor, reliability, and consistency. The use of Rogers’ DoI theory [14] adds analytical depth by providing a conceptual framework for understanding how factors (i.e., relative advantage, compatibility, complexity, trialability, and observability) influence the diffusion of EPAs in non-clinical educational contexts. This theoretical lens, supported by the comparison presented in Table 1, will help generate insights into the adaptation and scalability of EPAs to diverse non-clinical educational contexts.

Despite these strengths, certain limitations should be acknowledged. Although the search strategy is comprehensive, it is limited to English-language publications and publicly available materials, which may exclude relevant international or non-indexed studies. Additionally, although this review aims to explore the use of EPAs beyond medical education, with a focus on non-clinical educational contexts, the search strategy is primarily anchored in health and education-focused databases and incorporates search terms and filters derived from health professions education literature. As a result, the review may preferentially capture studies from health or education domains, while under-representing applications of EPAs or analogous frameworks in domains such as engineering, business, or other non-clinical fields. To mitigate this, grey literature sources and reference list screening will be used to identify relevant studies beyond traditional disciplinary boundaries. However, some domains may remain underrepresented. Notably, the concentration of findings within health-related contexts may itself reflect the current stage of EPA diffusion, highlighting areas where further cross-disciplinary exploration and application are needed. This potential bias should be considered when interpreting the breadth and generalizability of the findings. Additionally, the synthesis and interpretation of findings will depend on the level of contextual detail reported in included studies, potentially introducing interpretive bias when applying the DoI theory. Given the expected heterogeneity of literature, particularly across disciplines with limited documentation of EPA utilization, the generalizability of the findings may be constrained by variations in institutional structures, supervision models, and regulatory environments. Finally, the resource-intensive nature of the review, requiring multiple reviewers, iterative discussions to build consensus, and extensive data charting, may pose logistical challenges and extend the project’s timeline. Recognizing these constraints ensures transparency and supports appropriate interpretation of the review’s findings.

## Expected outcomes

By systematically examining the existing literature, this scoping review aims to map the diffusion of EPAs beyond medical education, identify how they have been adapted, developed, and implemented across curricula, examine their potential scalability to non-clinical educational contexts, and determine where EPAs may most appropriately diffuse in the future. Rogers’ DoI theory will serve as the interpretive lens for analyzing patterns within the findings, offering a means to understand the factors that enable or constrain the adoption of EPAs to non-clinical educational contexts. The findings from this review may provide recommendations to guide EPA-based assessment framework design and inform the development of policies, guidelines, and standards in non-clinical educational contexts. These practical outcomes hold relevance for involved parties (i.e., institutional administration, faculty, and trainees outside clinical professions), as the insights may guide the design of assessment frameworks that reliably develop and assess EPA entrustability and support workforce readiness upon graduation, addressing pressing challenges in CBE. Further, by identifying barriers and facilitators to the scaling and application of EPAs in non-clinical educational contexts, this review provides foundational guidance for institutions seeking to implement EPAs in these contexts, contributing to reliable competency assessment and the broader expansion of this innovative educational approach beyond medical education.

## Supporting information

S1 Checklist PRISMA-ScR checklist. (DOCX)

S2 Table Search strategy for Ovid MEDLINE. (DOCX)
