# Supplementary material for: Exploring the diffusion, application, and scalability of entrustable professional activities beyond medical education: A scoping review protocol
Source: PLoS One. 2026 Oct 22;21(10):e0330549. doi: 10.1371/journal.pone.0330549 (PMC13690217; doi:10.1371/journal.pone.0330549)
Supplement: Supplementary file 2 [file pone.0330549.s002.docx]

**S2 Table. Search strategy for Ovid MEDLINE**

| **Line #** | **Code** |
| --- | --- |
| 1 | entrustable professional activit*.mp. |
| 2 | entrustment.mp. |
| 3 | 1 or 2 |
| 4 | non-clinical program*.mp. |
| 5 | health professional education*.mp. |
| 6 | health professions education*.mp. |
| 7 | HPE.mp. |
| 8 | health sciences education*.mp. |
| 9 | (facilitator* or director* or teacher* or educator*).mp. |
| 10 | students, Health Occupations/ or students, dental/ or students, nursing/ or students, pharmacy/ or students, premedical/ or students, public health/ |
| 11 | ((audiology or or chiropract* or dental or dentistry or dietetics or health or health care or healthcare or law* or midwifery or nurs* or optometr* or paramedic* or pharmac* or psycholog* or public health or technolog* or therapy) adj3 (intern or interns or internship* or learner* or resident* or residency or student* or trainee* or training* or education*)).mp. |
| 12 | 10 or 11 |
| 13 | education, predental/ or education, premedical/ or exp education, dental/ or exp “education, biological and biomedical sciences, graduate”/ or education, dental, graduate/ or education, nursing, graduate/ or education, pharmacy, graduate/ or exp education, nursing/ or exp education, pharmacy/ or education public health professional/ or education, veterinary/ or exp internship, nonmedical/ |
| 14 | 4 or 5 or 6 or 7 or 8 or 9 or 12 or 13 |
| 15 | 3 and 14 |
